# Indole and 3-indolylacetonitrile inhibit spore maturation in *Paenibacillus alvei*

**DOI:** 10.1186/1471-2180-11-119

**Published:** 2011-05-27

**Authors:** Yong-Guy Kim, Jin-Hyung Lee, Moo Hwan Cho, Jintae Lee

**Affiliations:** 1 School of Chemical Engineering, Yeungnam University, Gyeongsan, Gyeonsangbuk-do 712-749, Korea

## Abstract

**Background:**

Bacteria use diverse signaling molecules to ensure the survival of the species in environmental niches. A variety of both Gram-positive and Gram-negative bacteria produce large quantities of indole that functions as an intercellular signal controlling diverse aspects of bacterial physiology.

**Results:**

In this study, we sought a novel role of indole in a Gram-positive bacteria *Paenibacillus alvei *that can produce extracellular indole at a concentration of up to 300 μM in the stationary phase in Luria-Bertani medium. Unlike previous studies, our data show that the production of indole in *P. alvei *is strictly controlled by catabolite repression since the addition of glucose and glycerol completely turns off the indole production. The addition of exogenous indole markedly inhibits the heat resistance of *P. alvei *without affecting cell growth. Observation of cell morphology with electron microscopy shows that indole inhibits the development of spore coats and cortex in *P. alvei*. As a result of the immature spore formation of *P. alvei*, indole also decreases *P. alvei *survival when exposed to antibiotics, low pH, and ethanol. Additionally, indole derivatives also influence the heat resistance; for example, a plant auxin, 3-indolylacetonitrile dramatically (2900-fold) decreased the heat resistance of *P. alvei*, while another auxin 3-indoleacetic acid had a less significant influence on the heat resistance of *P. alvei*.

**Conclusions:**

Together, our results demonstrate that indole and plant auxin 3-indolylacetonitrile inhibit spore maturation of *P. alvei *and that 3-indolylacetonitrile presents an opportunity for the control of heat and antimicrobial resistant spores of Gram-positive bacteria.

## Background

In nature, a key element of the adaptive responses of bacteria is the ability to sense and respond to the local environment, such as nutritional limitation, their population, the presence of toxic chemicals from other bacteria and host signals. Hence, it is important to coordinate the pattern of gene expression, and bacteria have evolved specific mechanisms to ensure the survival of the species in environmental niches. For example, many bacteria use a variety of intercellular signaling systems including quorum sensing. The intercellular signal molecules include *N*-acyl-homoserine lactones (AHLs) in Gram-negative bacteria, autoinducer 2 (AI-2) and indole in both Gram-negative and Gram-positive bacteria, signal peptides in Gram-positive bacteria, and others; these have been seen to co-ordinate gene expression for bioluminescence, sporulation, plasmid conjugal transfer, competence, virulence factor production, antibiotic production, and biofilm formation [1].

Indole is an intercellular signal [2,3] as well as an interspecies signal [4]. A variety of both Gram-positive and Gram-negative bacteria (more than 85 species) [2] produce indole using tryptophanase (TnaA; EC 4.1.99.1) that can reversibly convert tryptophan into indole, pyruvate, and ammonia according to reaction below [5].

Indole plays diverse biological roles in the microbial community; for example, indole controls the virulence [6-8], biofilm formation [4,9-11], acid resistance [4], and drug resistance [3,8,12,13] in Gram-negative bacteria. In a Gram-positive *Stigmatella aurantiaca*, indole increases its sporulation via indole binding pyruvate kinase [14,15]. Moreover, recent studies suggest that abundant bacterial indole in human intestines plays beneficial roles in the human immune system [16,17]. Also importantly, indole increases *Escherichia coli *antibiotic resistance, which eventually leads to population-wide resistance [3].

*P. alvei *(formerly known as *Bacillus alvei*) belongs to the class *Bacillales*, which includes *Bacillus*, *Listeria*, and *Staphylococcus *and is an endospore-forming Gram-positive bacterium that swarms on routine culture medium. *P. alvei *is frequently present in cases of European foulbrood (a disease of the honey bee) [18] and has, on occasion, been the cause of human infections [19-21]. *P. alvei *is the only indole-producing bacterium among many *Bacillus *species [22], and the biosynthesis of indole has been well-studied in *P. alvei *[22-24]. It has long been thought that indole producing bacteria including *P. alvei *utilize tryptophanase to synthesize tryptophan and other amino acids from indole as a carbon source [24,25]. However, the equilibrium of the reaction favors the production of indole from tryptophan [26,27]. Hence, we sought here the real biological role of indole in *P. alvei *physiology.

Spore-forming bacteria can respond to nutritional limitation and harsh environmental conditions by forming endospores that are remarkably resistant to heat, desiccation, and various chemicals [28,29]. Spore formation is an elaborate and energy intensive process that requires several hours to complete [29]. Therefore, sporulation is a last-resort adaptive process that is tightly regulated by complex cell-cell signaling or so-called quorum sensing [29,30]. *Bacillus subtilis *produces multiple cell-cell signaling molecules to control the sophisticated sporulation [30] that is often a temporal, spatial, and dynamic decision-making process [28].

The outermost protective layers of *B. subtilis *endospores are the coat and the cortex [31]. The spore coat is a barrier against bactericidal enzymes and destructive chemicals. Therefore, heat resistant spores are also resistant to treatment by various chemicals, such as acids, bases, oxidizing agents, alkylating agents, aldehydes and organic solvents [32]. Thus, we investigated the role of indole on heat resistance as well as other environmental stresses.

In this study, we identified that indole was a stationary phase extracellular molecule in *P. alvei *and functioned to inhibit spore maturation and to decrease survival rates under several environmental stresses. Additionally, we studied the effect of indole derivatives originated from plants on spore formation in *P. alvei*. This study provides another important role of indole and indole derivatives.

## Results

### Extracellular indole accumulation in *P. alvei*

**Figure 1:**
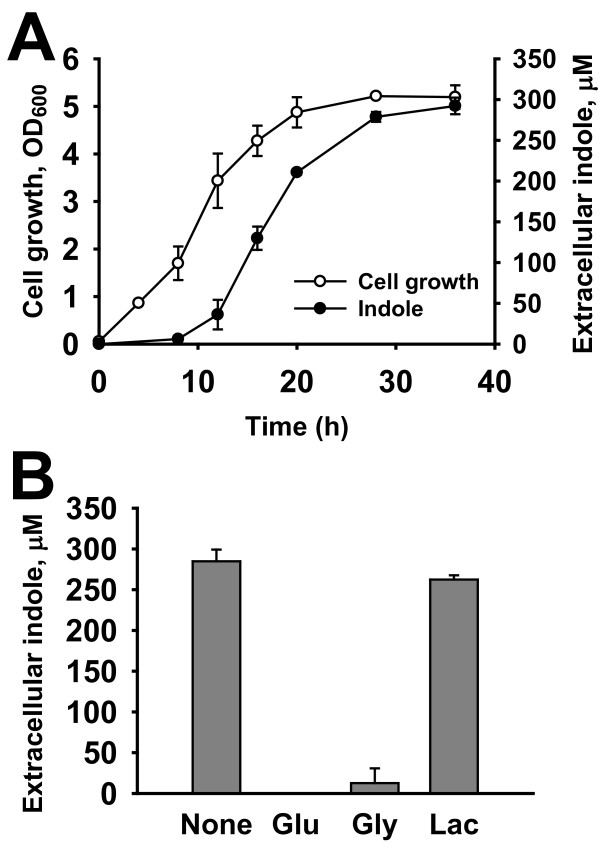
**Production of extracellular indole in *P. alvei***. Cell growth and extracellular indole accumulation in LB (A) and extracellular indole accumulation in LB supplemented with different carbon sources (B) at 37°C at 250 rpm. Cell growth (closed circle) was determined via the optical density at 600 nm (OD_600_). Glucose (Glu), glycerol (Gly), and lactose (Lac) in 0.5% (w/v) were added at the beginning of the culture and cells were cultured for 36 h and indole production was measured. Experiments were performed in triplicate and one standard deviation is shown.

To be an environmental signal molecule, indole has to be excreted out of cells. Thus, the cell growth of *P. alvei *and the extracellular indole concentration were measured in Luria-Bertani (LB) medium. Clearly, the level of extracellular indole from *P. alvei *was growth-dependent (Figure 1A). Indole production was begun in the middle of exponential growth phase and reached the maximum amount (300 μM) in the stationary phase. Notably, the level of extracellular indole present was stable over time at 37°C (Figure 1A), which was one of characteristics of the indole molecule [2] while other signaling molecules, such as AHLs, AI-2, and signal peptides, are only temporally present and heat-unstable [2]. The accumulation pattern of extracellular indole was similar to that of other bacteria, such as *E. coli *[33] and *Vibrio cholera *[10], while these two bacteria accumulated up to 500-600 μM of extracellular indole within 24 h in LB [10,33]. The slower accumulation of indole in *P. alvei *was probably due to the 200-fold lower activity of *P. alvei *tryptophanase than that of *E. coli *tryptophanase [22].

### Catabolite repression of *P. alvei *tryptophanase

Since indole production was suppressed by the presence of glucose in *E. coli *through catabolite repression [34], the effect of carbon sources on indole production was investigated in *P. alvei*. Similar to *E. coli*, the addition of glucose and glycerol (0.5%) in LB medium completely abolished the production of indole in *P. alvei *for 36 h, while lactose (0.5%) did not affect indole accumulation (Figure 1B). This result suggested that the indole accumulation in *P. alvei *was strictly controlled by catabolic repression although transport mechanisms of glucose and glycerol would be different. In other words, *P. alvei *did not produce indole in the presence of the preferred carbon sources such as glucose and glycerol. Unlike the current observation, it was previously reported that the tryptophanase in *B. alvei *(renamed as *P. alvei*) appeared to be constitutive, and catabolite repression was not operative [22]. The report studied the effect of only tryptophan on tryptophanase activity and found that the activity of *P. alvei *tryptophanase was independent of tryptophan [22].

### Indole inhibits the heat-resistant cell numbers of *P. alvei*

**Figure 2:**
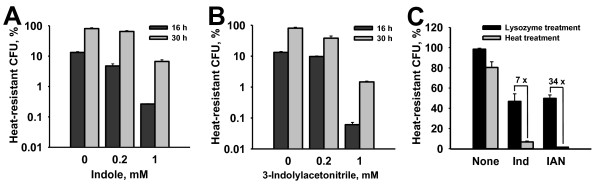
**Effect of indole and 3-indolylacetonitrile on the heat-resistant CFU of *P. alvei***. The cells (an initial turbidity of 0.05 at 600 nm) were grown in spore forming DSM medium for 16 h and 30 h. Exogenous indole (A) and 3-indolylacetonitrile (B) were added at the beginning of the culture to test the effect of indole (Ind) and 3-indolylacetonitrile (IAN) on the heat-resistant CFU. Lysozyme-resistance assays (C) were performed with 30 h-grown cells with and without indole and 3-indolyacetonitrile, and lysozyme (1 mg/mL) was treated for 20 min. Each experiment was repeated three to four times and one standard deviation is shown.

The main hypothesis of this study was that a large quantity of extracellular indole would play a quorum sensing role in cell physiology of *P. alvei *so we investigated the effect of indole on sporulation and biofilm formation which was influenced by cell population and environmental stresses in other *Bacillus *strains [30]. In *P. alvei*, the addition of exogenous indole (0, 0.2, or 1.0 mM) surprisingly decreases the heat-resistant colony-forming unit (CFU) in a dose dependent manner (Figure 2A). For example, indole (1 mM) decreased the heat-resistant CFU of *P. alvei *compared to no addition of indole 51-fold at 16 hr (0.26 ± 0.01% vs.13.2 ± 0.9%) and 10-fold at 30 hr (8 ± 6% vs. 77 ± 10%). To confirm the presence of exogenous indole, the indole level in DSM medium was measured with HPLC. The level of exogenous indole (1 mM) was not changed at all over 24 h (data not shown). Hence, the exogenous indole was not utilized as a carbon source and inhibited the heat-resistant CFU of *P. alvei*.

Additionally, the temperature effect of indole on the heat resistance of *P. alvei *was investigated since the environmental temperature affected indole signaling in *E. coli *[12]. Unlike in *E. coli*, the inhibitory effect of indole (1 mM) on the heat-resistant CFU of *P. alvei *at 30°C (0.3 ± 0.1% vs. 8 ± 2% for 16 h) was similar to that at 37°C in *P. alvei *(Figure 2). Hence, it appeared that the temperature effect of indole on the heat-resistant CFU of *P. alvei *was not significant under the tested laboratory conditions.

### Indole inhibits the development of spore coat and cortex

**Figure 3:**
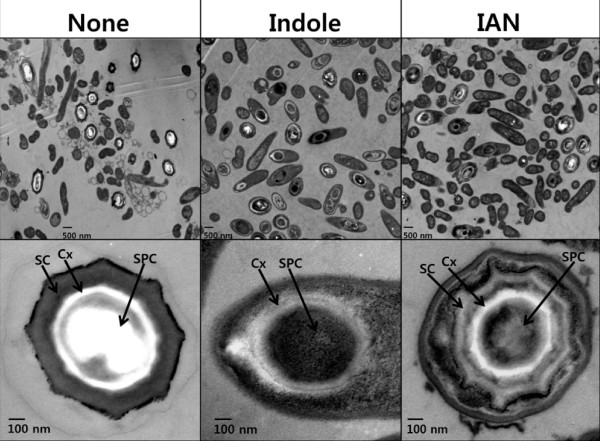
**Electron microscopy analysis of *P. alvei *endospore formation**. DMSO (0.1% v/v) was used as a control (None). 1 mM indole and 1 mM 3-indolylacetonitrile (IAN) dissolved in DMSO were added at the beginning of culture, and cells (an initial turbidity of 0.05 at 600 nm) were grown in DSM for 30 h. The scale bar indicates 500 nm in the upper panel and 100 nm in the lower panel. Abbreviations: SC, spore coat; Cx, cortex; SPC, spore core.

The effect of indole on the morphology of sporulating cells was examined by transmission electron microscopy. Surprisingly, the proportion of sporulating cells in the total number of cells was similar between with and without treatment of indole (upper panel in Figure 3). However, exogenous addition of indole influenced the morphology of the spore coat and the cortex. Cells with exogenous indole formed endospores with a thin spore coat and a thin spore cortex, while using no indole treatment resulted in a thick spore coat and cortex (lower panel in Figure 3). Because the spore coat and cortex were important for heat resistance and chemical resistance [31], we concluded that indole caused an immature spore that negatively contributed to the heat resistance of *P. alvei*.

### Effect of indole derivatives on the heat resistance of *P. alvei*

In the natural environment, indole can be easily oxidized into hydroxyindoles by diverse oxygenases, and indole derivatives often show different effects on bacterial physiology [2]. Thus, *P. alvei *can often encounter many kinds of indole-like compounds that are synthesized from tryptophan in other bacteria, plants, and even animals. Therefore, seven indole derivatives have been further investigated for the heat resistance of *P. alvei*.

**Figure 4:**
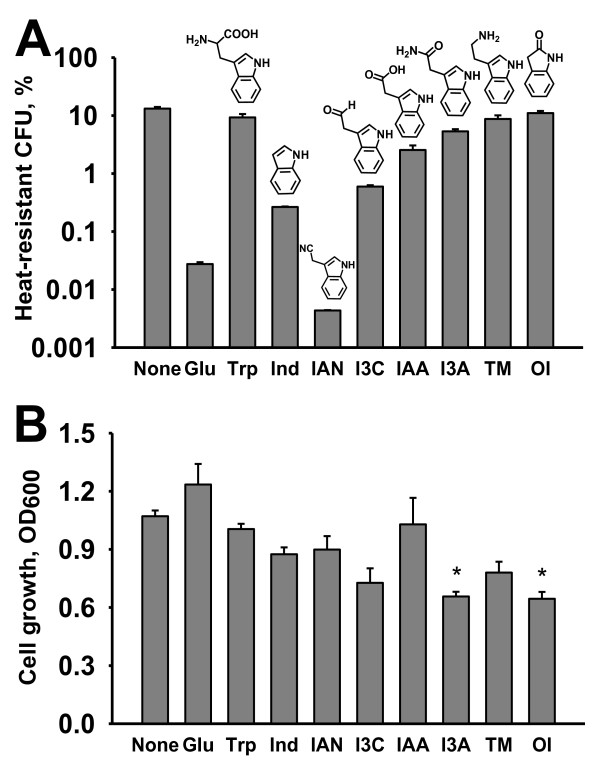
**Effect of indole derivatives on the heat-resistant CFU of *P. alvei***. The cells (an initial turbidity of 0.05 at 600 nm) were grown in spore forming DSM medium for 16 h. Exogenous indole derivatives (1 mM) and glucose (0.5% w/v) were added at the beginning of the culture. Tryptophan (Trp) was dissolved in water, and indole (Ind), 3-indolylacetonitrile (IAN), indole-3-carboxyaldehyde (I3C), 3-indoleacetic acid (IAA), indole-3-acetamide (I3A), tryptamine (TM), and 2-oxindole (OI) were dissolved in dimethyl sulfoxide (DMSO). DMSO (0.1% v/v) was used as a control (None). Each experiment was repeated three to four times and one standard deviation is shown. The structures of Trp, Ind, IAA, I3CA, IAN, I3A, TM, and OI are shown. The asterisk indicates statistical significance determined using a Student *t *test (P < 0.05).

As a negative control, glucose was used since glucose decreased the sporulation of *B. subtilis *[35]. Similar to *B. subtilis*, glucose (0.5%) clearly decreased the heat-resistant CFU by 600-fold in *P. alvei *(Figure 4A). However, L-tryptophan as the main substrate of the indole biosynthesis did not have much influence on the heat-resistant CFU, which supported that indole rather than tryptophan specifically influenced the heat resistance of *P. alvei *(Figure 4A).

Most interestingly, a plant auxin, 3-indolylacetonitrile dramatically (up to 2900-fold) decreased the heat-resistant CFU of *P. alvei *in a dose dependent manner at 16 and 30 hr (Figure 2B and Figure 4A), while another auxin 3-indoleacetic acid had a less significant influence, and tryptamine and 2-oxindole had no effect (Figure 4A). Therefore, these results suggest that the functional groups of indole derivatives may control the development of *P. alvei *spores.

Similar to indole, the proportion of sporulating cells in the total number of cells was similar with and without treatment of 3-indolylacetonitrile (upper panel in Figure 3). Also, 3-indolylacetonitrile produced an irregular spore coat, while no treatment produced sturdy coat (Figure 3). Therefore, it appeared that indole and 3-indolylacetonitrile inhibited spore maturation rather than sporulation initiation.

In order to understand how most spores (upper panel in Figure 3) in the presence of indole and 3-indolylacetonitrile could not survive against heat treatment, the lysozyme resistance assay [36] was performed with 30-hour grown cells since the lysozyme treatment could release all spores. As a result, indole and 3-indolylacetonitrile produced a large portion of lysozyme-resistant cells (47 ± 8% with indole and 50 ± 3% with 3-indolylacetonitrile) which are probably the number of total spores, while indole and 3-indolylacetonitrile produced only 6.7 ± 0.9% and 1.5 ± 0.1% heat-resistant cells (Figure 2C); hence it appeared that a large number of spores have some spore defect for heat resistance. Therefore, it appeared that the low heat-resistant CFU was caused by some spore defect or the altered spore structure.

Furthermore, the effect of indole and 3-indolylacetonitrile was investigated using another spore-forming medium, Brain Heart Infusion (BHI) agar for a longer incubation time (here, 14 days) when sporulation process would be completed. Similar to DSM medium, indole (1 mM) and 3-indolylacetonitrile (1 mM) inhibited the heat-resistant CFU of *P. alvei *(17 ± 10% and 16 ± 1%), compared to no addition of exogenous indole (77 ± 3%). Therefore, the inhibitory impact of indole and 3-indolylacetonitrile was effective in different media for a long term, while their effect on heat resistance was attenuated with a longer incubation time.

### Effect of indole and indole derivatives on cell growth

To test the toxicity of indole and indole derivatives, cell turbidity at 16 hr and the specific growth rates with indole and 3-indolylacetonitrile were measured. Most indole derivatives at the concentration tested (1 mM) did not have much of an inhibition effect on the cell growth of *P. alvei*, while indole-3-acetamide and 2-oxindole (P < 0.05) slightly decreased cell growth (Figure 4B). The growth rate of *P. alvei *was 1.38 ± 0.08/h in the absence of the indole derivatives in LB medium, whereas the growth rate was 1.30 ± 0.01/h with indole (1 mM) and 1.27 ± 0.01/h with 3-indolylacetonitrile (1 mM). In DSM medium, the growth rate of *P. alvei *was 0.19 ± 0.01/h in the absence of the indole derivatives, whereas the growth rate was 0.17 ± 0.01/h with indole (1 mM) and 0.15 ± 0.01/h with 3-indolylacetonitrile (1 mM). Therefore, indole and 3-indolylacetonitrile were not toxic to *P. alvei *and the inhibitory effect of the heat resistance was mostly due to the function of indole and 3-indolylacetonitrile rather than growth inhibition.

### Indole contributes to low survival against environmental stresses

**Figure 5:**
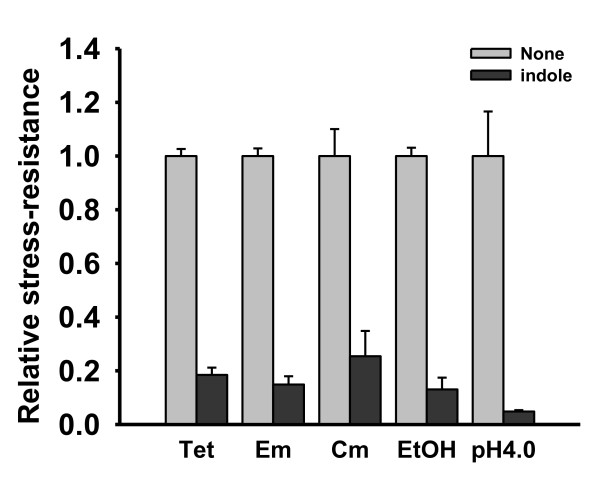
**Effect of indole on stress-resistance of *P. alvei***. The cells (an initial turbidity of 0.05 at 600 nm) were grown in spore forming DSM medium for 16 h. After the 16 h incubation, cells (1 ml) were placed in contact with antibiotics, 70% ethanol, and pH 4.0 LB for 1 h. Tet, Em, and Cm stand for tetracycline (1 mg/ml), erythromycin (5 mg/ml), and chloramphenicol (1 mg/ml), respectively. EtOH and pH 4.0 stand for 70% ethanol and pH 4.0 LB, respectively. Each experiment was repeated two to four times and one standard deviation is shown.

Since endospores are remarkably resistant to heat as well as various chemicals [28,29], we presumed that indole also decreased the resistance to environmental stresses, such as treatment with antibiotics, ethanol and low pH. As expected, indole decreased the survival rates with three antibiotics (tetracycline, erythromycin, and chloramphenicol) and when exposed to low pH and 70% ethanol (Figure 5). For example, indole decreased tetracycline resistance 5.4-fold, erythromycin resistance 6.7-fold, and chloramphenicol resistance 4-fold, and the survival rates with ethanol 8.5-fold and pH 4.0 21-fold, respectively. These results are a good match with the sporulation results (Figure 2).

### Effect of indole on the survival of *B. subtilis *spores

**Figure 6:**
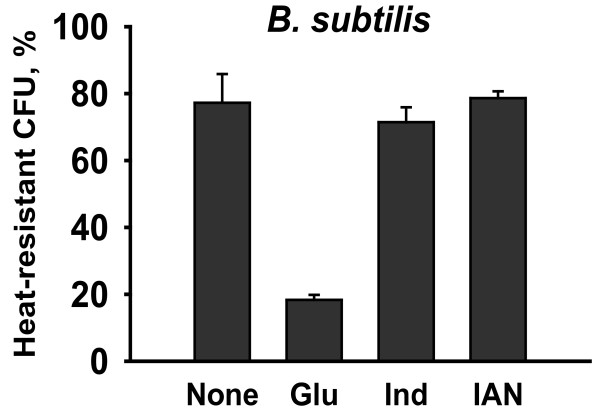
**Effect of indole and 3-indolylacetonitrile on the heat-resistant CFU of *B. subtilis***. Glucose (0.5% w/v), indole (1 mM) and 3-indolylacetonitrile (1 mM) were added at the beginning of culture, and cells (an initial turbidity of 0.05 at 600 nm) were grown in spore forming DSM medium at 37°C for 16 h. Glucose (Glu) was dissolved in water, and indole (Ind) and 3-indolylacetonitrile (IAN) were dissolved in DMSO. DMSO (0.1% v/v) was used as a control (None). DMSO (0.1% (v/v)) alone did not affect cell growth and the heat-resistant CFU. Each experiment was repeated three to four times and one standard deviation is shown.

Since *P. alvei *belongs to the same *Bacillales *order including *B. subtilis *(the most studied spore-forming bacterium), the effect of indole and 3-indolylacetonitrile was investigated in *B. subtilis *that did not produce indole (data not shown). Unlike *P. alvei*, indole and 3-indolylacetonitrile had no impact on the heat resistance in *B. subtilis*, while glucose treatment as a negative control significantly decreased the heat-resistant CFU (Figure 6). Hence, it appeared that the action mechanism of indole was different between indole-producing *P. alvei *and non-indole-producing *B. subtilis*.

## Discussion

Indole is an abundant environmental signal in both Gram-positive and Gram-negative bacteria [2]. Currently, the diverse roles of indole as an intercellular signal are beginning to be revealed in various indole-producing-bacteria, such as *E. coli *[2,3], *Vibrio cholerae *[10], *Stigmatella aurantiaca *[14,15], *Fusobacterium nuceatum *[11], and *Porphyromonas gingivalis *[37], as well as in non-indole-producing bacteria, such as *Pseudomonas aeruginosa *[8] and *Salmonella enterica *[13,38]. The current study shows that the environmental signal indole also has a role in Gram-positive *P. alvei*.

Interestingly, the role of indole seems to be substantially divergent in different microorganisms, reflecting adaptation to different environments and niche-specific challenges. For example, indole differently controls (increases or decreases) biofilm formation in different *E. coli *strains [2], *Vibrio cholerae *[10], and *Fusobacterium nuceatum *[11]. Also, indole and indole derivatives induced sporulation in *Stigmatella aurantiaca *[14], while this study shows that indole reduced the integrity of spores in *P. alvei *(Figure 3). Therefore, the results suggest that different bacterial species have developed their unique systems to beneficially utilize indole in their microbial community.

Previously, it was reported that indole derivatives, such as 3-indoleacetic acid, 3-indolylacetonitrile, tryptamine, and 2-oxindole, but not indole, decreased the percentages of spore germination and appressorium formation, which inhibited all stages of infection behaviors in a rice pathogen *Magnaporthe grisea *[39]. These results and the current study suggest that indole derivatives, such as 3-indolylacetonitrile, can be used as protective compounds against spore-forming *P. alvei*.

Since indole influenced the biofilm formation of several indole-producing bacteria, such as *E. coli *[2], *Vibrio cholerae *[10], and *Fusobacterium nuceatum *[11], and the sporulation transcription factor SpoA was required for biofilm development in *B. subtilis *[40], the effect of indole on the biofilm formation of *P. alvei *was investigated. However, indole did not show an effect on *P. alvei *biofilm formation in the 96-well plate biofilm assay in LB or DSM media either at 30°C and at 37°C (data not shown). Therefore, the indole-involving mechanism of *P. alvei *biofilm formation is different from that in other strains.

Glucose obviously prevented the development of CFU of *P. alvei *presumably by preventing sporulation (Figure 4) as well as in *B. subtilis *via catabolite repression [35]. Because indole is a stationary phase signal (Figure 1A) and because the production of indole is tightly regulated by carbon sources (Figure 1B), the role of indole on spore formation could be closely affected by the catabolite repression. Thus, indole serves not only as an indicator of cell population, but also as an indicator of starvation. This dual function of indole may reflect the status of cells in the environment. Because the accumulation of extracellular indole can be dramatically affected by many environmental factors (pH, temperature, and the presence of antibiotics) in addition to carbon sources [41], the action of indole would be governed by the environment in a sophisticated manner.

Nevertheless, the question remains as to why *P. alvei *produces copious amount of extracellular indole, as it causes immature spore formation (Figure 3). One possible explanation can be found in the previous study in that bacteria utilize indole as a defense tool against non-indole producing pathogenic *P. aeruginosa *to diminish its virulence [8]. Another possible answer is that indole intentionally lowers integrity of spores in order to make cells easy to resume growth when the environment is favorable again at a later date. Hence, a large quantity of indole is an indicator of a favorable environment in which other unfavorable species are scare and indole may control the timing of germination in natural environments. Although highly speculative, another possibility is that indole signal negatively controls spore maturation, while other quorum sensing molecules positively regulates sporulation of *Bacillus*, even using multiple signaling molecules [30]. Also, there is the possibility that indole is affecting spore germination since indole lowered the survival against environmental stresses (Figure 5) while the number of spore was not affected by indole (Figure 3).

However, it is unclear, so far, how the indole signal influences sporulation in *P. alvei*. It is necessary to identify the operon of *P. alvei *tryptophanase to understand the genetic regulation of indole biosynthesis. For further transcriptional study, the *P. alvei *chromosome should be sequenced. Also, one of future work would be to study which stage of the sporulation cascade or what genetic mechanism is being affected by indole. For example, it is interesting to find indole-interacting proteins in *P. alvei*, as previously identified indole-binding PykA of *S. aurantiaca *[15].

Endospore formation is an altruistic behavior of mother cells that provides the maximum chance of survival for the group (daughter cells) over any its neighbor species [28]. However, the formation of an environmentally resistant spore of pathogenic bacteria, such as *Bacillus anthracis *and various *Clostridium *app., are problematic to human health [28]. Hence it is important to find a tool which controls sporulation as a disinfectant or sporocide. The current study has revealed the natural action of sporulation reduction by indole and the plant auxin 3-indolylacetonitrile. Previously, 3-indolylacetonitrile from cruciferous vegetables (*Brassica*), such as broccoli, cauliflower, and cabbage, was seen to decrease the biofilm formation of two pathogenic bacteria, *E. coli *O157:H7 and *P. aeruginosa *by inhibiting polymeric matrix production [42]. Hence, indole and 3-indolylacetonitrile are possible spore maturation inhibitors against spore-forming *P. alvei *and biofilm inhibitors against pathogenic biofilm formation. Currently, various indole derivatives from plants and numerous synthetic indole derivatives are commercially available and work is in progress to identify universal and stronger sporocides and to understand their genetic mechanism in action.

## Conclusions

The current study demonstrates that i) indole is an extracellular stationary phase molecule in a Gram-positive bacteria *P. alvei*, ii) indole clearly inhibits spore maturation and survival rates under several stresses in *P. alvei *without affecting cell growth, iii) plant auxin 3-indolylacetonitrile dramatically decreased the heat resistance of *P. alvei*, iv) electron microscopy shows that indole and 3-indolylacetonitrile inhibit the development of spore coats and cortex in *P. alvei*. This study shows that indole, as a signaling molecule in quorum-sensing manner, plays a role in sporulation of *P. alvei *and that 3-indolylacetonitrile can be useful to control of heat and antimicrobial resistant spores of Gram-positive bacteria.

## Methods

### Bacterial strains, materials and growth rate measurements

*P. alvei *(ATCC 6344) and *B. subtilis *strain (ATCC6633) were obtained from Korean Culture Center of Microorganisms. The strain was originally isolated from European foulbrood [43]. Luria-Bertani (LB) [44] was used as a basic medium for growth unless indicated. DSM medium (Difco sporulation medium [45]) was used for spore formation and cell survival tests with antibiotics. DSM medium contains 8 g of Bacto nutrient broth (Difco), 10 ml of 10% KCl, 10 ml of 1.2% MgSO_4_·7H_2_O, 1.5 ml of 1 M NaOH, 1 ml of 1 M Ca(NO_3_)_2_, 1 ml of 0.01 M MnCl_2 _and 1 ml of 1 mM FeSO_4 _per liter. BHI agar medium (Difco brain heart infusion agar) was also used for long-term spore formation. Indole, tryptophan, 3-indoleacetic acid, indole-3-carboxyaldehyde, 3-indolylacetonitrile, indole-3-acetamide, tryptamine, 2-oxindole, tetracycline, erythromycin, chloramphenicol, and streptomycin were purchased from Sigma-Aldrich Co. (Missouri, USA). Ethanol and dimethyl sulfoxide (DMSO) were purchased from Duksan Pure Chemical Co. (Ansan, Korea). Bacterial strains were initially streaked from -80°C glycerol stocks on LB plates, and a fresh single colony was inoculated into LB medium (25 ml) in 250 ml flasks and routinely cultured at 250 rpm at 37°C unless otherwise indicated. Overnight cultures were diluted in a 1:100 ratio using LB medium for cell growth and indole production or DSM medium for the test of spore surviving. For cell growth measurements, the optical density was measured at 600 nm (OD_600_) with a spectrophotometer (UV-160, Shimadzu, Japan). When the value of OD_600 _was above 0.7, the culture sample was diluted to fit within a linear range of between 0.2 and 0.7. In order to measure cell viability and cell number, diluted cells were enumerated with LB agar plates.

### Indole assays

To measure the concentration of extracellular indole, *P. alvei *was grown in LB medium at 250 rpm for 36 h. The extracellular indole concentrations were measured with reverse-phase HPLC [4] using a 100 × 4.6 mm Chromolith Performance RP-18e column (Merck KGaA, Darmstadt, Germany) and elution with H_2_O-0.1% (v/v) trifluoroacetic acid and acetonitrile (50:50) as the mobile phases at a flow rate of 0.5 ml/min (50:50). Under these conditions, the retention time and the absorbance maximum were 5.1 min/271 nm for indole. Each experiment was performed with three independent cultures.

### Sporulation assay

Sporulation assays were performed in the spore-forming DSM medium and on BHI agar plates. The overnight culture of *P. alvei *grown in LB was diluted in a 1:100 ratio in DSM and then re-grown to a turbidity of 0.5 at 600 nm. The cells were re-inoculated in a 1:10 ratio in DSM (an initial turbidity of 0.05 at 600 nm) and grown for 16 hr and 30 hr at 30°C and 37°C. To test the effect of indole and indole derivatives on the heat-resistant CFU, the indole or indole derivatives were added at the beginning of the culture in DSM medium. After incubation for 16 hr and 30 hr, aliquots of each culture (1 ml) were incubated in a water bath at 80°C for 10 min [46], the cells were then immediately diluted with phosphate buffer (pH 7.4) to cool down, and then the cells were enumerated with LB agar plates. To study the long-term effect of indole and indole derivatives, BHI agar was used and the previous assay [47] was adapted. The percentage of heat-resistant cells was calculated as the number of CFU per ml remaining after heat treatment divided by the initial CFU per ml at time zero. Since glucose decreased sporulation rate in *B. subtilis *via catabolite repression [35], glucose was used as a negative control.

### Stress resistance assays

All survival assays were performed in DSM medium as the sporulation assay. In order to test the effect of indole and indole derivatives, indole or 3-indolylacetonitrile (1 mM) were added at the beginning of the culture in DSM, and the cells were grown for 16 h in DSM. After the incubation, four antibiotics (tetracycline at 1 mg/ml, erythromycin at 5 mg/ml, and chloramphenicol at 1 mg/ml) were mixed with the cells (1 ml) and incubated at 37°C for 1 h without shaking, and then cells were enumerated with LB agar plates. To determine the impact of indole on ethanol resistance and acid resistance, 16 h-grown cells were mixed with 70% ethanol and LB (pH 4.0) and incubated at 37°C for 1 h without shaking, and cells were enumerated with LB agar plates. For lysozyme-resistance assays, 30 h-grown cells with and without indole and 3-indolyacetonitrile were treated with lysozyme (1 mg/mL) in buffer (20 mM Tris-HCl [pH 8.0], 300 mM NaCl) and incubated at 37°C for 20 min [36]. Aliquots of serial dilutions in PBS buffer were then spotted on LB agar plates to determine the number of survivors. Each experiment was performed with three independent cultures.

### Crystal-violet biofilm assay

A static biofilm formation assay was performed in a 96-well polystyrene plate (Fisher Scientific, Pittsburg, USA) as previously reported [48]. Briefly, cells were inoculated at an initial turbidity at 600 nm of 0.05 and incubated for 24 h without shaking at both 30°C and 37°C. Cell density (turbidity at 620 nm) and total biofilm (absorbance at 540 nm) were measured using crystal violet staining.

### Transmission electron microscopy (TEM)

To examine the spore structure, TEM was used and a previous method [49] was modified. Briefly, *P. alvei *cells were grown in DSM as performed in sporulation assays. After culturing *P. alvei *cells with and without indole or 3-indolylacetonitrile for 30 h, 2.5% glutaraldehyde and 2% formaldehyde were added to pre-fix the cells and incubated overnight at 4°C. Then, cells were collected by centrifugation and post-fixed in 2% osmium tetroxide overnight at 4°C, and washed four times with 0.2 M phosphate buffer (pH 7.2). Then, cells were mixed with warm 2% agarose and polymerized. Cell block was sliced into 0.5 × 0.5 × 0.1 cm, dehydrated with ethanol and embedded in Epon resin (Hatfield, USA). Ultrathin sections were obtained using a MT-X ultramicrotome (Tucson, USA) and stained with 3% uranyl acetate. TEM images were obtained using a Hitachi H-7600 electron microscope (Tokyo, Japan).

## Authors' contributions

YK carried out most of the experiments and helped to draft the manuscript. J-HL participated in the design of study and interpretation of the data. MHC participated in discussion of the study. JL conceived of the study, participated in its design and coordination, and wrote much of the manuscript. All authors read and approved the final manuscript.
